# MicroRNAs as Promising Therapeutic Agents Against Prostate Cancer Resistant to Castration—Where Are We Now?

**DOI:** 10.3390/pharmaceutics16111347

**Published:** 2024-10-22

**Authors:** Mariana Ferreira, Mariana Morais, Rui Medeiros, Ana Luísa Teixeira

**Affiliations:** 1 Molecular Oncology and Viral Pathology Group, Research Center of IPO Porto (CI-IPOP)/RISE@CI-IPOP (Health Research Network), Portuguese Oncology Institute of Porto (IPO Porto)/Porto Comprehensive Cancer Center (Porto.CCC), 4200-072 Porto, Portugal; mariana.f.ferreira@ipoporto.min-saude.pt (M.F.); mariana.gomes.morais@ipoporto.min-saude.pt (M.M.); ruimedei@ipoporto.min-saude.pt (R.M.); 2 ICBAS, Abel Salazar Institute for the Biomedical Sciences, University of Porto, 4050-313 Porto, Portugal; 3 Biomedical Research Center (CEBIMED), Faculty of Health Sciences, Fernando Pessoa University (UFP), 4249-004 Porto, Portugal; 4 Research Department, LPCC-Portuguese League Against Cancer (NRNorte), 4200-172 Porto, Portugal; 5 Faculty of Medicine (FMUP), University of Porto, 4200-319 Porto, Portugal

**Keywords:** miRNA mimics, miRNA inhibitors, castration-resistant prostatic cancer

## Abstract

MicroRNAs are a conserved class of small, tissue-specific, non-coding RNAs that regulate gene expression to preserve cellular homeostasis. Proper miRNA expression is crucial for physiological balance because it affects numerous genetic pathways, including cell cycle control, proliferation, and apoptosis, through gene expression targeting. Deregulated miRNA expression has been implicated in several cancer types, including prostate cancer (PC), acting as tumor suppressors or oncogenes. Despite the availability of promising therapies to control tumor growth and progression, effective diagnostic and therapeutic strategies for different types of cancer are still lacking. PC continues to be a significant health challenge, particularly its castration-resistant (CRPC) form, which presents major therapeutic obstacles because of its resistance to conventional androgen deprivation treatments. This review explores miRNAs’ critical roles in gene regulation and cancer biology, as well as various miRNA delivery systems, highlighting their potential and the challenges in effectively targeting cancer cells. It aims to provide a comprehensive overview of the status of miRNA research in the fight against CRPC, summarizing miRNA-based therapies’ successes and limitations. It also highlights the promise of miRNAs as therapeutic agents for CRPC, underlining the need for further research to overcome existing challenges and move these therapies toward clinical applications.

## 1. Introduction

Over 30 years ago, the first microRNA (miRNA) was discovered in the nematode *Caenorhabditis elegans* (*C. elegans*) through the identification of the development regulator lin-4 [1]. Initially, it was believed to be a conventional gene encoding proteins; however, it was later discovered that lin-4 did not code for a protein but for a regulatory RNA consisting of 22 nucleotides [2]. This discovery, though surprising, was limited to the *C. elegans* research community. Later, a second miRNA, let-7, was identified [3]. This finding was highly significant as let-7 is conserved across many organisms, emphasizing its central role in the biology of this small class of RNAs [3]. Currently, over 30,000 mature miRNAs have been identified across 206 species, with 2588 miRNAs present in the human genome alone [4]. Collectively, these miRNAs are thought to regulate approximately one-third of the genes in the human genome [4].

MiRNAs are short, single-stranded RNA molecules, typically 18–25 nucleotides in length, serving as master regulators of gene expression through their function as guide molecules for post-transcriptional gene regulation in various organisms [5]. Each miRNA regulates the expressions of hundreds of target genes, reinforcing the crucial role of the miRNA pathway as an essential mechanism for the control of gene expression [6]. The level of complementarity between miRNAs and their target genes dictates the mode of the gene suppression. Full matching has been demonstrated to trigger mRNA cleavage, while partial matching results in translational inhibition [7].

MiRNA production involves a series of steps beginning with transcription in the nucleus and ending with processing and maturation in the cytoplasm [8,9]. RNA polymerases II or III first transcribe primary miRNA transcripts (pri-miRNAs), which have a hairpin-like structure containing the sequence for the mature miRNA [8,9]. The microprocessor complex, which includes the RNase III enzyme Drosha and the DGCR8 protein, identifies and cleaves these pri-miRNAs, producing precursor miRNAs (pre-miRNAs), with a hairpin shape [8,9]. These pre-miRNAs are then transported to the cytoplasm by exportin 5 (XPO5) [8,9]. In the cytoplasm, the RNase III endonuclease Dicer further processes the pre-miRNAs by recognizing specific features at their 5′ end and the 3′ overhang near the terminal loop, resulting in a short, double-stranded RNA duplex [8,9]. This duplex consists of the mature miRNA strand (the guide strand) and its complementary strand (miRNA*) [8,9]. Dicer, assisted by the transactivation-responsive RNA-binding protein (TRBP), enhances its cleavage activity through helicase interaction [8,9]. TRBP also facilitates the binding of Dicer to Argonaute proteins (AGO2), which promotes the formation of the miRNA-induced silencing complex (RISC). Within the RISC complex, the leader strand is retained, while the passenger strand is typically degraded [8,9]. The mature miRNA within the RISC then binds to target mRNA molecules via complementary base pairing [8,9]. Depending on how well the miRNA matches its target mRNA, it can either cause mRNA degradation or inhibit its translation. High complementarity generally leads to mRNA degradation, whereas partial complementarity leads to translational repression without mRNA degradation [8,9].

MiRNAs have been reported to bind to various regions, such as the open reading frame (ORF) or 5′ untranslated region (UTR) of mRNA, as well as gene promoters [10]. These small molecules perform essential regulatory functions in several cellular processes, including development, differentiation, metabolism, proliferation, invasion, migration, apoptosis, and genomic stability [6,11]. This occurs through precise base-pairing interactions with target transcripts, as well as through the transcriptional and post-transcriptional regulation of their expressions [10].

Over the last decades, the dysregulation of miRNA expression has been observed in diverse human diseases, such as metabolic diseases and cancer [12,13]. The deregulation of miRNAs in cancer can be because of epigenetic changes, alterations in miRNA biogenesis, polymorphisms or mutations in the sequences that encode for these miRNAs, or chromosomal abnormalities. These factors can also occur together, leading to aberrant expressions of specific miRNAs in cancer cells [14,15]. The first identification of a cancer linked to miRNA was documented in 2002 [16].

About two decades ago, Calin and coworkers made a revolutionary discovery regarding miRNA deregulation in cancer, more specifically, in chronic lymphocytic leukemia (CLL) [16]. Although deletion on chromosome 13q14 is associated with most cases of B-cell CLL, efforts to identify tumor suppressor genes present in this region have been unsuccessful. Calin and coworkers demonstrated that the genetic sequences encoding miR-15 and miR-16 were located within a 30 kb loss in CLL and that these miRNAs were deleted or downregulated in the vast majority of cases of CLL patients [16]. Later, Cimmino and colleagues described that miR-15 and miR-16 target B-cell lymphoma protein 2 (Bcl2), an antiapoptotic protein overexpressed in CLL [17]. The negative regulation of these miRNAs resulted in an increase in Bcl2 protein levels [17]. Since then, research has increasingly revealed the dysregulated expressions of miRNAs in multiple tumor types [18,19,20].

Indeed, the observed aberrant miRNA expressions in different tumor models have been shown to influence one or several of the cancer hallmarks proposed by Hanahan and Weinberg, thereby contributing to tumor initiation and progression [21].

In fact, miRNAs can be classified as oncomiRs or tumor suppressor miRNAs, regarding their function and expression deregulation in cancer [22]. OncomiRs, characterized by their overexpression in tumors, act by suppressing tumor suppressor mRNAs, thus promoting the proliferation and metastasis of tumor cells [22]. One example is miR-21. High levels of miR-21 have been documented in the sera, plasmas, and tumor tissues of patients with pancreatic, prostate, stomach, lung, and ovarian cancers, and its inhibition was associated with a reduction in cancer proliferation and reversed drug resistance in pancreatic, ovarian, and breast cancers [23,24,25,26,27,28,29]. Additionally, the aberrant expression of miR-21 can contribute to hepatocellular carcinoma (HCC) growth and spread by modulating PTEN expression, further highlighting its role in tumor progression [30]. Moreover, miR-221/222 is another oncomiR that is reported as upregulated in several cancers [31]. The elevated expression of miR-221/222 has been documented in liver tumorigenesis, where it promotes cancer cell growth by inhibiting the CDK inhibitor p27, and in the plasmas of patients with renal cell carcinoma [31,32]. In breast cancer, it modulates tumor development and progression by influencing signaling pathways. Additionally, miR-221/222 overexpression is observed in colon and pancreatic tumors [33].

In contrast, tumor suppressor miRNAs are known to inhibit cancer progression. Reduced expressions of these molecules play critical roles in the processes of cancer development and proliferation [15]. The let-7 family of miRNAs functions as a tumor suppressor, acting as a regulatory influence on key oncogenes, such as Myc, Rasa, MYCN, and high mobility group A2 (HMGA2) [34,35]. In lung cancer cells, the ectopic expression of let-7 induces cell death, enhancing its essential role in fighting cancer progression [36]. Beyond lung cancer, let-7 also functions as a tumor suppressor in breast cancer, preventing malignant cell growth mediated by Erα [37]. Its multifaceted actions against various oncogenes underscores the significance of let-7 as a key agent in inhibiting tumorigenesis across different types of cancer. Another family that has emerged as an essential player in several cancers is the mir-29 family, which has the potential to regulate more than 4000 gene products [38]. In the respiratory system, it has been implicated in lung adenocarcinoma, lung cancer, and non-small cell lung cancer [38]. In the nervous system, miR-29 is reported to be involved in both neuroblastoma and glioblastoma, while its impact extends to the digestive system, with roles in HCC, colon cancer, stomach cancer, and esophageal cancer [38,39,40,41]. Additionally, it influences the muscular and skeletal systems, particularly in osteoblastoma, and is important in the reproductive and genitourinary systems, contributing to bladder cancer, ovarian cancer, and prostate cancer [38]. Dysregulation of miR-29 is consistently linked to tumor progression and invasion in these various cancers, highlighting its tumor-suppressive capabilities [38].

MiRNAs’ relevance in the oncology context has raised the interest of the scientific community regarding their biomarker potential. Indeed, the molecular characterization of tumors has been proved to be significant in improving diagnosis, patient follow-up, and monitoring of therapy [42]. However, the invasive process of conventional tissue biopsy and the challenges of obtaining enough quantity and quality to profile the tumor prevent effective monitoring, especially given the heterogeneity of the tumor and metastatic spread [43]. The liquid biopsy approach arose to overcome these limitations, focusing on the analysis of the tumor content in biological fluids, such as cerebrospinal fluid, urine, mucosa, pleural effusions, and blood [44]. This approach is based on the presence of tumor cells or derivatives of these cells in these biological fluids and provides increased sensitivity using a minimally invasive methodology, enabling the collection of repeated samples and facilitating the monitoring of the response to treatment and the identification of recurrences [45].

MiRNAs present several characteristics that show their potential as biomarkers. On one hand, miRNAs can circulate in biofluids, either within protein complexes, such as the RNA-induced silencing complex, where they bind to argonaut 2 (AGO2), or within extracellular vesicles [46]. These mechanisms protect miRNAs from degradation by RNAse, making them highly stable under adverse conditions and reinforcing their potential as biomarkers [47]. On the other hand, their deregulation has been shown repeatedly in the oncology context.

The deregulation of miRNA expression is a common occurrence in cancer, leading to distinct expression profiles that may contribute to the early detection of the disease. Wang and collaborators have screened 735 miRNAs from the sera of prostate cancer patients, from which miR-1290 showed the most promising diagnostic performance when compared to healthy individuals [48]. Additionally, serum levels of miR-1290 demonstrated the ability to differentiate between healthy individuals and patients with early-stage pancreatic cancer [48]. Notably, miR-1290 outperformed CA19-9, a conventional diagnostic marker with a sensitivity ranging from 70% to 80% in the sera of prostate cancer patients [49]. Moreover, various studies have pointed out the prognostic and predictive importance of circulating miRNAs associated with cancer because as cancer progresses to a more invasive phenotype, miRNAs undergo changes, serving as molecular markers of tumor cells, observable from tumorigenesis to subsequent stages of progression [50,51]. Consequently, circulating miRNAs have emerged as highly reliable candidates for monitoring the disease. Serum levels of miR-200c were shown to be significantly increased during colorectal cancer metastasis [52]. A high level of serum miR-200c showed a significant correlation with the lymph nodes and distant metastasis, thereby suggesting that serum miR-200c levels could be used as an indicator for predicting colorectal metastasis [52]. MiR-375 and miR-200b were shown to be significantly upregulated in the sera of patients with metastatic prostate cancer compared with patients with localized cancer [53]. In recent studies, elevated serum levels of miR-17-5p/20a and miR-21 in gastric cancer patients were shown to be significantly associated with gastric cancer metastasis, implying that this miRNA pair may serve as a diagnostic or prognostic marker for this disease [54,55].

Circulating miRNAs are very promising as biomarkers for cancer diagnosis, but many studies are merely reporting changes in miRNA levels in the plasmas/sera of cancer patients [52,53,55]. The lack of standardized procedures for sample preparation, RNA isolation, and selection of internal controls makes it difficult to compare results between laboratories [56]. The development of reference protocols for the quantification of circulating miRNA is imperative for future research. Furthermore, assessing the relationship between circulating miRNAs and established cancer markers is crucial for translational purposes. Understanding the origin of circulating miRNAs in both healthy individuals and cancer patients, as well as the factors that influence their levels and roles in cancer pathology, requires in-depth research. This knowledge not only establishes circulating miRNAs as reliable cancer biomarkers but also paves the way for new therapeutic strategies.

## 2. miRNAs: Promising Therapeutic Strategies in Cancer Treatment

Among the plethora of cancer treatments, none offers the complete eradication of cancer cells, and they are often combined with questionable side effects. However, advances in understanding the molecular mechanisms of cancer have allowed for us to target cells at their root. The abnormal expressions of several carcinogenesis-associated miRNAs occur during tumor initiation and progression, suggesting that the manipulation of miRNA expression may hold promise as a potential strategy for cancer treatment [57,58].

The most recent approach to miRNA therapy revolves mainly around two strategies: the enhancement or reconstitution of endogenous miRNAs that act as pathological suppressors and the expressional reduction or functional blocking of miRNAs that act as pathological drivers [59]. This dual strategy offers a promising avenue for the development of effective treatments that counteract the deregulated miRNA expression patterns observed in cancer cells.

**Figure 1:**
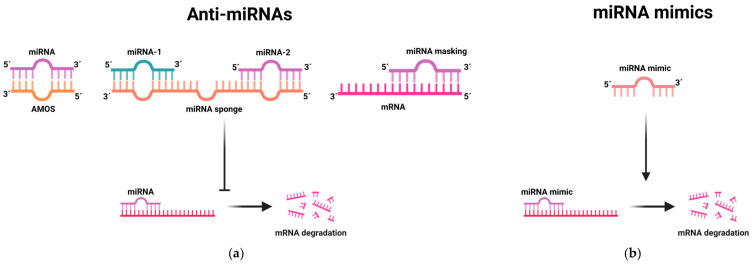
MiRNA-based therapies: (**a**) Different types of anti-microRNAs; (**b**) miRNA mimics. Created with BioRender.

Oncogenic miRNAs, normally overexpressed in tumors, need to be suppressed to their normal levels. This homeostasis restoration allows for their target genes, mainly tumor suppressors, to become active, inhibiting tumorigenesis or progression [22]. Anti-miRs, which are synthesized to target endogenous miRNAs, typically consist of single-stranded oligonucleotides with complementarity to the endogenous miRNA sequence [60]. Therapeutic strategies against oncogenic miRNAs encompass several approaches, including anti-miRNA oligonucleotides, miRNA sponges, small-molecule inhibitors, and miRNA masking, as seen in Figure 1a.

Anti-miR oligonucleotides are short strands of chemically modified nucleic acids, typically including 17–22 nucleotides [59]. These molecules are specifically engineered to match and bind to target miRNAs, effectively blocking the interaction between the mature miRNA and its target gene. By binding to miRNA, anti-miRs disrupt its function, thereby preventing its regulatory effects on gene expression [59]. Anti-miR oligonucleotides are frequently modified chemically to increase their stability. In a study conducted on *C. elegans*, modified anti-miR oligonucleotides with 2′O-methyl modifications, designed to be complementary to let-7 miRNA, resulted in the loss of the let-7 function [61]. MiRNA sponges function as competitive inhibitors equipped with numerous binding sites for a specific endogenous miRNA, with the aim of preventing its interaction with the target mRNA [62]. Their effectiveness in repressing target miRNAs is comparable to that of anti-miR oligonucleotides. These sponges can be adapted with complementary heptamer seeds, allowing for a single sponge to target an entire family of miRNA seeds [62]. Research conducted by Ma et al. used miRNA sponges to suppress miR-9 expression in breast cancer cells, resulting in a significant inhibition of metastasis formation through the upregulation of CDH1 [63]. MiRNA masking consists of designing sequences that perfectly complement the miRNA binding site on the mRNA. These sequences form strong duplex structures with the target mRNA, effectively blocking the binding site from interacting with the miRNA [60]. The urgent search for miRNA-based inhibition therapies has also stimulated the creation of small molecules capable of preventing miRNA biogenesis or disrupting miRNA–target interactions [60]. A recently published study by Hei et al. described the development of fluoroquinolone derivatives designed to function as miR-21 inhibitors [64]. However, the stability and efficacy of these small-molecule inhibitors should be evaluated.

Tumor suppressor miRNAs, in contrast, are frequently downregulated in cancer, leading to the upregulation of their target oncogenes [22]. The administration of the exogenous expression of tumor suppressor miRNAs offers a strategy to restore their decreased levels, with the purpose of impeding the cellular pathways leading to oncogenesis. MiRNA mimics (Figure 1b) serve as an effective way for restoring the functionality of tumor suppressor miRNAs. These mimics are small double-stranded RNA molecules chemically modified with 2′-O′methoxy groups to resemble endogenous mature miRNA molecules [65]. MiR-34 is a well-established tumor suppressor miRNA, acting on numerous cellular pathways relevant to cancer, such as the p53 and Wnt/β-catenin pathways [66]. In various types of tumors, including lung, liver, breast, and colon carcinomas, miR-34 is typically downregulated [66]. However, studies have shown that reintroducing its function through exogenous miR-34 mimetics effectively prevents tumor growth and progression [66].

### miRNA Delivery System

**Figure 2:**
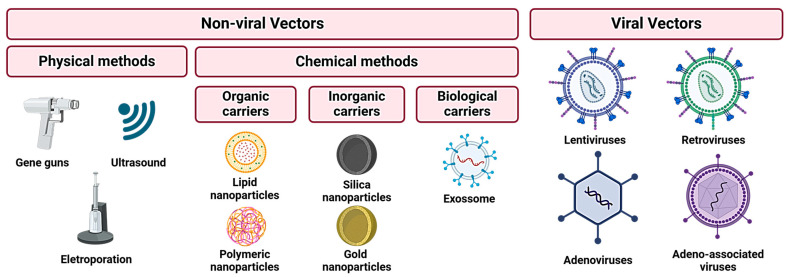
Different types of miRNA delivery systems. Created with BioRender.

The main challenge in translating miRNA therapies into clinical practice lies in the lack of a reliable delivery system (Figure 2). Despite their small size, miRNAs cannot passively diffuse through lipid membranes [67]. In addition, their limited biological stability is another obstacle to effective delivery. Ideally, miRNA delivery vehicles should have a high loading capacity, good stability, enhanced half-life in circulation, slow degradation of the miRNA cargo, and minimal toxicity [67]. Nowadays, available options for the effective delivery of miRNA include non-viral vectors and viral vectors [68,69].

An efficient non-viral delivery system is distinguished by its ability to transport endogenous miRNA or vectors expressing miRNA, preventing nuclease-mediated degradation [69]. This delivery process can be performed by physical methods, such as electroporation, gene guns, or ultrasound or by chemical methods using organic, inorganic, or polymer-based carriers [69]. Non-viral systems usually have lower toxicity and immunogenicity compared to their viral equivalents but with a slightly reduced transfection efficiency [69]. Organic-based therapies use liposomes, which are, by far, the most popular approach [70]. This method involves combining lipids with cationic head groups along with helper lipids, some of which may incorporate polyethylene glycol chains to mask the surface charge [69]. Wu et al. designed an effective cationic liposome to restore levels of the tumor suppressor miRNA miR-29 in non-small cell lung cancer. Through the systemic administration of the liposome–miRNA complex, they were able to restore miR-29 levels in cancer cells, subsequently preventing tumor growth [71]. Gold nanoparticles, silica-based nanoparticles, and Fe_3_O_4_-based nanoparticles can also be used for miRNA delivery, with gold nanoparticles being the most commonly used ones [72,73,74].

Naturally derived polymer-like cell-penetrating peptide (CPP) and synthetic polymers, such as polyethylenimine (PEI), poly(lactide-co-glycolide) (PLGA), and poly(amidoamine) (PAMAMs) dendrimers, are used in polymer-based delivery systems. The successful delivery of miRNA (miR-29b) has been accomplished using CPP in osteogenic stem cells [75]. PLGA, an FDA-approved biodegradable polymer, was employed in the delivery of miR-26a to HepG2 cells through PLGA nanoparticles that were modified with a polyplexed PEI coating [72]. PAMAMs are positively charged polymers known for their high transfection efficiency and have been utilized for delivering anti-miR-21 to glioblastoma cells, effectively inhibiting miR-21 activity [69,74]. Exosomes have also been used to encapsulate and deliver synthetic or endogenously expressed miRNAs [76]. The oligonucleotide cargo can be introduced by transfection of the corresponding plasmid into exosome-producing cells, or synthetic oligonucleotides can be inferred through electroporation of the mature exosomes [76].

Genetically engineered viruses offer a versatile platform for the delivery of specific oligonucleotides, allowing for the positive regulation of miRNA expression or the delivery of anti-miRs to inhibit the expression of the target miRNA [77]. Various viral vectors, including lentiviruses, retroviruses, adenoviruses, and adeno-associated viruses, are used in viral-based miRNA delivery systems [69].

Retroviral vectors, derived from members of the Retroviridae family, use RNA viruses with genome sizes ranging from 7 to 11 KB. They can carry genetic sequences and integrate them into the host cell’s genome during mitosis, which makes them suitable for dividing cells [78]. Retroviral vectors proved to be effective in inducing miR-138 expression in mouse embryonic fibroblasts [79]. Lentiviruses, a subgroup of retroviruses, are distinct in their ability to infect both dividing and non-dividing cells. Systemic in vivo lentiviral administration of miR-15a/16 was found to decrease malignancy in a mouse model of chronic lymphocytic leukemia [80]. Adenoviruses, double-stranded DNA viruses, can carry large genes but are unable to integrate exogenous genes into the host genome [69]. Adeno-associated viruses, single-stranded DNA viruses, accommodate genetic sequences compatible with miRNA genes and have the ability to infect dividing and non-dividing cells, expanding their usability [81]. These innovative miRNA therapeutic systems present a promising avenue for novel therapeutics, offering a unique approach to cancer treatment by modulating entire biological pathways.

Development in the understanding of the roles of miRNAs together with the optimized efficacy and safety of anti-miR/miRNA-mimic-based strategies are contributing to the transposition of miRNA research into clinical practice [82]. Currently, several RNA-based drugs have been authorized by the FDA for medical use, and the therapeutic possibilities of mRNA treatments are gaining more attention in a wide range of human diseases, leading to the daily expansion of clinical trials of mRNA-based therapies [83]. Despite significant progress in preclinical research, the field of miRNA-based therapeutics is still at an early stage of development. Table 1 highlights that only a limited number of miRNA therapies for cancer have entered clinical trials, with none having progressed to phase III or Food and Drug Administration (FDA) approval. Moreover, several of these therapies are encountering termination because of concerns regarding toxicity.

Although there are several challenges to overcome in advancing therapeutic miRNAs for clinical use, these barriers can be identified distinctly and effectively addressed. Vector encapsulation, bioavailability, the selection of the best target, the efficiency of delivery systems to target organs, immunogenicity, toxicity, off-target binding, and the development of more effective modified ASOs continue to challenge the possibility for translating miRNA-based approaches into therapeutic realities [84,85]. Many other critical issues need to be addressed before miRNA therapeutics can be widely adopted as novel clinical treatments, and further research is needed to determine their efficacy as therapeutic targets or therapeutic agents for clinical applications. Nevertheless, their potential applications in different tumor models raises increasing interest in the scientific community, including in prostate cancer.

Prostate cancer (PCa) is a leading cause of cancer-related death. Despite the advantages of local therapy and initial hormone therapy, most patients will eventually experience a relapse, developing castration-resistant prostate cancer (CRPC), associated with a poor prognosis [86]. For this reason, the search for new strategies is urgently needed.

**Table 1:** Clinical trials with miRNA therapeutic agents in cancer treatment.

MiRNA Drug	Mode of Action	Phase	Clinical Trial Number(s) *	Status	Targeted miRNA	Disease/Condition	Reference(s)
MRX34	miRNA mimic	Phase I	NCT01829971	Terminated	miR-34a	Primary liver cancer; Melanoma; NSCLC; SCLC; Renal cell carcinoma; Lymphoma; Multiple myeloma	[87]
MesomiR 1	miRNA mimic	Phase I	NCT02369198	Completed	miR-16	Non-small cell lung cancer; Malignant pleural mesothelioma	[88,89,90]
Cobomarsen/ MRG-106	Anti-miR	Phase I Phase II Phase II	NCT02580552 NCT03713320, NCT03837457	Completed Terminated Terminated	miR-155	Mycosis fungoides; Cutaneous T-cell lymphoma; Chronic lymphocytic leukemia; Diffuse large B-cell lymphoma; ABC-subtype Adult T-cell leukemia/lymphoma	[91,92]
miR-10	anti-miR-10	Observational	NCT01849952	Recruiting	miR-10	Glioma	--
miR-10b	Usage of miR-10b for diagnostic purposes and evaluation of anti-miR-10b in vitro as a therapy option	Observational	NCT01849952	Recruiting	miR-10b	Astrocytoma; Anaplastic astrocytoma; Oligodendroglioma; Anaplastic oligodendroglioma; Oligoastrocytoma; Anaplastic oligoastrocytoma; Glioblastoma; Brain tumors; Brain cancer	--
MRX34	miRNA mimic	Phase I Phase II	NCT02862145	Withdrawn (5 immune-related severe adverse events in Phase I)	miR-34a	Solid tumors	[87]
INT-1B3	miRNA mimic	Phase I	NCT04675996	Recruiting	miR-193a-3p mimic	Advanced solid tumors	--
Serum MicroRNA-25	miR-25 for diagnostic purposes	Observational	NCT03432624	Not yet recruiting	miR-25	Pancreatic cancer	--

* NCT-numbered trials are registered at ClinicalTrials.gov.

## 3. Prostate Cancer

Prostate cancer is a heterogeneous disease that affects middle-aged men between the ages of 45 and 60, being one of the most incident cancers in the men worldwide [93]. In 2022, GLOBOCAN reported 1,467,854 new cases of PCa, which represent 14.2% of all cancers in men [94].

According to the grade, stage of development, age, and performance status, different therapeutic approaches can be applied. The stage of the PCa, determined at the first diagnosis, is defined on the bases of the cancer’s extension, serum PSA levels, and the ISUP grading system [95]. The main treatment options for the disease at an early stage include surgery (prostatectomy), radiotherapy, and brachytherapy [95]. In locally advanced or metastatic cancers, androgen deprivation therapy (ADT), chemotherapy, and radiotherapy seem to be the most effective treatment strategies [95].

ADT affects the androgen pathway because both androgens and androgen receptors play critical roles in the growth and progression of PCa. [96]. This treatment has shown excellent results, achieving disease remission in approximately 90% of patients. However, in the long term, they develop resistance to ADT and progress to castration-resistant prostate cancer (CPRC), which is characterized by a few therapeutic options, a poor prognosis, and an impaired life quality [97]. As a result, the need for the development of new therapies has emerged [98]. In recent years, several therapeutic options have been approved for the treatment of CRPC, including poly ADP ribose polymerase (PARP) inhibitors, chemotherapeutic agents, agents that target bone metastases, and second-generation antiandrogens [99]. Despite the availability of several approved treatment options, they only extend overall survival by a few months, making the development of new therapeutic agents crucial for improving patients’ qualities of life.

Emerging evidence suggests that a promising potential approach to treat PC involves targeting overexpressed miRNAs through anti-miRNA therapies while simultaneously increasing the expressions of underexpressed miRNAs through the administration of mimics [100]. In fact, several deregulated miRNAs are involved in PC progression, CRPC development, and metastasis formation. In the context of tumor progression, miR-1 and miR-99b are examples of noteworthy miRNAs. MiR-1 is downregulated in PC cells and has been shown to reduce cell viability and proliferation by targeting the c-Met/AKT/mTOR signaling pathway [101]. Similarly, miR-99b, another tumor suppressor miRNA, is frequently downregulated and affects differentiation, proliferation, invasion, and migration in PC [102]. In the development of CRPC, miR-221-3p is significantly downregulated, particularly in bone metastases, which contributes to disease progression and increased metastatic potential [103]. Additionally, miR-23c and miR-4328 are downregulated during metastasis, suggesting a loss of their tumor-suppressive effects [104]. Conversely, certain miRNAs, such as miR-20b-5p and the miR-183-96-182 cluster, are upregulated and associated with aggressive PC phenotypes and poor prognoses [105].

Indeed, the value of miRNAs as therapeutic agents in the context of cancer has been extensively studied. However, little is known about the use of these molecules as therapeutic agents in CRPC and the consequences of restoring their expression levels, as well as their target mRNAs, in disease progression. Therefore, in this review, we summarize the use of mimics/anti-miRNAs of deregulated miRNAs in CRPC, as well as the potentially deregulated target proteins.

## 4. Evidence Acquisition

**Figure 3:**
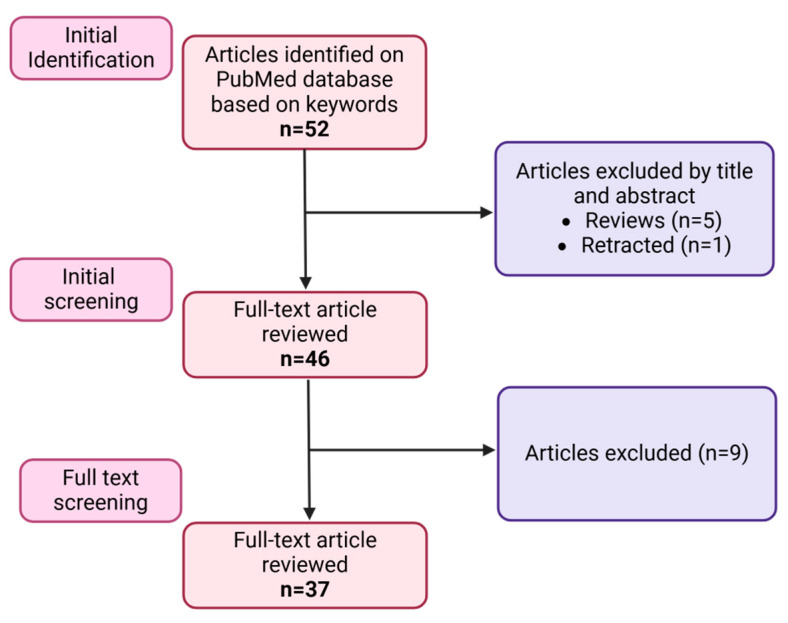
Diagram of evidence acquisition.

We performed a literature search on the PubMed database, using key terms such as “miRNA mimics”, “miRNA inhibitors”, and “Prostatic cancer castration resistant”. All the papers published until 2 August 2024 were included. The search yielded 52 papers based on the combination of these keywords. Among them, five were review articles, and one was retracted. After the analysis, nine papers were excluded, as they either did not discuss the use of miRNA mimics/inhibitors (eight) or we did not have access to the full text (one). Subsequently, the remaining 37 full papers underwent detailed analysis and review (Figure 3).

## 5. Evidence Synthesis

To analyze the information from all the articles considered, the data were primarily organized according to the name of the miRNA drug, the type of assay (in vitro/in vivo/ex vivo), the target protein, and the delivery method. The data were divided into two tables based on whether miRNA mimics or inhibitors were transfected (Table 2 and Table 3, respectively). Table 2, which focuses on the use of miRNA mimics, shows that most studies have used liposomal delivery systems to transfect miRNAs into cell and animal models. On the other hand, Table 3 summarizes studies focusing on the use of miRNA inhibitors to suppress the activity of oncogenic miRNAs. The studies highlighted in this table often used inhibitors or antisense oligonucleotides to inhibit miRNAs that are frequently overexpressed in prostate cancer. Additionally, Supplementary Tables S1 and S2 are organized according to the name of the miRNA drug, the targeted miRNA, the levels of miRNA expression, and the key outcomes obtained from each study, with Supplementary Table S1 focusing on the use of mimics and Supplementary Table S2 on inhibitors, mirroring the organization of Table 2 and Table 3.

These tables highlight the diversity of miRNA-based therapeutic strategies that currently exist and underscore the importance for selecting specific targets and appropriate delivery methods to optimize the treatment efficacy.

## 6. Discussion

Patients with PCa commonly develop resistance to ADT and progress to CRPC, which is associated with limited treatment options and a poor prognosis [97]. Despite the availability of treatment options, they only prolong survival by a few months, highlighting the need for new therapies [98]. Recently, miRNAs have received attention because of their deregulated expressions in cancers and their roles in cancer development, progression, and metastasis and resistance to therapy [143,144]. MiRNA-based therapies, used alone or in combination with other treatments, have shown promise, and there is already strong evidence that these therapies could be beneficial in the treatment of PCa [100].

Several preclinical studies have explored restoring the expressions of deregulated miRNAs in CRPC using in vitro, in vivo, and ex vivo models. Table 2 and Table 3 demonstrate the existence of several studies that are already in the in vivo phase, offering more relevant results that can be translated into possible clinical applications. This shows that the potential of these therapies has been widely explored in the fight against CRPC.

It is also noticeable that the transfection of mimics has prevailed compared to the transfection of miRNA inhibitors. This may be because transfecting mimics can be easier than transfecting miRNA inhibitors, in terms of stability and effectiveness in increasing the activities of specific miRNAs [145]. Furthermore, the fact that miRNA mimics have a more redundant sequence makes them less specific than miRNA inhibitors [146]. The development and production of miRNA mimics are also easier because inhibitors sometimes require additional adjustments to improve their efficacy and stability [146]. Finally, although the efficient delivery of both is still a challenge to be overcome, miRNA mimics tend to be more compatible with existing delivery systems, such as nanoparticles or viral vectors [147].

As mentioned above, the effectiveness of delivery systems in targeting specific organs remains a significant obstacle to the translation of miRNA-based approaches into therapeutic applications. [85]. Lipofectamine appears to be the transfection reagent of choice for most researchers. This may be due to not only the fact that lipofectamine has already been used widely in various research studies but also the various advantages that lipid-based systems have, such as the ability to be functionalized for targeting, the ability to co-deliver gene therapy and chemotherapy, high-capacity gene packaging, systemic gene delivery, controllable size, transient expression, and the fact that they are not immunogenic [148].

When comparing various therapeutic approaches, it is evident that certain miRNAs exhibit more significant therapeutic potential in CRPC. Zhu et al. reported that miR-149 may play a role in CRPC development by affecting Ras, Rho proteins, and the SCF complex, while Zhao et al. stated that modulating Akt expression levels through the overexpression of miR-149 may represent a potential novel therapeutic approach for CRPC [107,111]. Yu et al. discovered that N-Myc regulates the miR-421/ATM pathway differentially, contributing to ADT and enzalutamide resistance, and that the combined use of an ATM inhibitor and enzalutamide could resensitize N-Myc-overexpressing CRPC cells [127]. Yadav et al. found that by sequestering miR-421, MALAT1 shields PCa tumor cells from anticancer drugs by initiating the HR pathway [131]. These findings highlight the potential for targeting miRNA pathways involved in drug resistance, offering new avenues for combination therapies. Indeed, combining miRNA-based therapies with existing treatments, such as docetaxel and enzalutamide, may improve therapeutic outcomes, as miRNA modulation may increase drug sensitivity [109,117].

The connection between miRNAs and cancer stem cells (CSCs) also represents a critical therapeutic target. Jin et al. demonstrated that the tumor-suppressive effects of miR-128 are linked to its ability to inhibit CSCs by targeting key molecules, such as BMI-1, and Lo et al. mentioned that STAT1-IFIT5 plays a crucial role in PCSC acquisition by accelerating the turnover of specific microRNAs, such as miR-128 [118,141].

In fact, all these articles reinforce the crucial roles of miRNAs in the development of CRPC. Rönnau et al. associate high expression levels of miR-3687 with decreased cell migration and invasion, while high levels of miR-3195 increase cell migration [115]. Armstrong et al. showed how, in fact, the deregulation of miRNA levels is associated with resistance to therapies, demonstrating how the knockdown of miR-181a restores sensitivity to docetaxel in resistant cells, and Naiki-Ito et al. did the same by showing that the overexpression of miR-8080 allows for resistance to enzalutamide to be overcome [109,117]. Sun et al. also linked miR-542-3p to metastasis [123]. Wang et al. demonstrate that exosomal miR-26a can be used as a personalized delivery vehicle for the targeted delivery of therapeutic agents to prostate cancer cells and tissues [54]. Taken together, these studies suggest that miRNAs not only play pivotal roles in tumor suppression and resistance reversal but also represent a promising avenue for personalized medicine in CRPC.

Although considerable progress has been made in understanding the roles of miRNAs in the pathogenesis and progression of CRPC, significant obstacles remain to their translation into clinical practice. One of the main challenges is the intricate network of miRNA interactions with multiple oncogenic pathways, which makes it difficult to identify specific targets that can be manipulated for therapeutic benefit.

Moreover, the development of effective delivery systems for miRNA-based therapies remains a formidable hurdle. The stable and targeted delivery of miRNAs to tumor cells is essential to maximize therapeutic efficacy while minimizing systemic toxicity. Current delivery methods, including lipid nanoparticles, viral vectors, and exosomal carriers, are promising but far from ideal. Issues such as degradation by nucleases in the bloodstream, poor tissue specificity, and inefficient cellular uptake limit the full exploitation of miRNA-based therapies. Additionally, off-target effects pose a significant challenge, as miRNAs may inadvertently bind to unintended mRNA targets, leading to unwanted gene silencing in non-cancerous tissues, which could result in toxic side effects. 

However, to fully exploit the therapeutic potential of miRNAs, future research must prioritize overcoming current barriers to delivery and specificity. Advances in nanoparticle design, improved tissue-targeting strategies, and the development of novel carriers capable of delivering miRNAs with high precision will be critical for achieving clinical success. In addition, deeper insights into miRNA biology, particularly miRNAs’ context-dependent roles at different stages of CRPC, are needed to refine miRNA selection and ensure that therapeutic strategies are adapted to the unique molecular landscape of each patient’s tumor. Overcoming these challenges is essential for the successful translation of miRNA-based therapies from the bench to the bedside, potentially revolutionizing the treatment landscape for CRPC.

## 7. Conclusions

In just over 20 years since the first miRNA was identified, the field of miRNA biology has grown considerably. MiRNA-based therapies as potential treatments for cancer are at an advanced stage of validation, and some encouraging results have already been achieved. Despite the large number of preclinical studies conducted on miRNA therapeutics over the years, only a few have entered clinical development. This is mostly because there are still many challenges to overcome.

On one hand, one of the major challenges in the development of miRNA-based therapeutics is the identification of the most appropriate miRNA candidates or targets for each disease. One the other hand, designing delivery systems that enhance the stability of therapeutic agents, ensuring tissue-specific targeting, and minimizing potential toxicities and off-target effects are other key challenges that need to be addressed.

The challenges associated with the use of miRNA-based therapies need to be explored and overcome to fully realize the clinical potential of these therapies in the treatment of cancer, particularly CRPC, which is associated with few therapeutic options and a poor prognosis, given the promising results observed to date. The fast pace of discovery and innovative delivery, dosing, and target selection strategies, combined with a growing body of knowledge and extensive preclinical analysis using novel delivery platforms, place miRNA-based therapies in a position to become a key long-term solution in the fight against cancer.

## Figures and Tables

**Table 2:** MiRNA mimic studies in the CRPC context.

miRNA Drug(s)	Study Type	miRNA Target	Delivery Method	Reference(s)
miR-1247-5p	In vitro (PC-3 and LNCaP cell lines)	MYCBP2	DharmaFECT Duo transfection reagent (lipid-based reagent)	[106]
miR-218, miR-145, miR-197, miR-149, miR-122, and let-7b	In vitro (C4-2 cell line)	---	Lipofectamine3000 (Invitrogen, Waltham, MA, USA)	[107]
miR-1205	Ex vivo (clinical samples) In vitro (LNCaP cell line)	EGLN3	Lipofectamine RNAiMAX (Thermo Fisher Scientific, Waltham, MA, USA)	[108]
miR-181a	In vitro (C4-2B, C4-2B (docetaxel resistant), DU145, DU145, docetaxel-resistant and PC3 cell lines)	---	Lipofectamine2000 (Invitrogen, Waltham, MA, USA)	[109]
miR-30a	In vivo (Xenograft models) In vitro (22RV1 cell line)	MYBL2, FOXD1, and SOX4	Hiperfect transfection reagent (Qiagen, Hilden, Germany)	[110]
miR-149	In vitro (C4-2 cell line)	Akt1	Lipofectamine^®^ 2000 (Invitrogen; Thermo Fisher Scientific, Inc.)	[111]
miR-26a	In vitro (PC3 and LNCaP cell lines)	---	Lipofectamine^®^ 3000 reagent (Invitrogen)	[112]
Cy3-modified miR-222-3p	Ex vivo (clinical samples) In vitro (LNCaP cell line)	mTOR	Lipofectamine 2000 (Invitrogen)	[113]
miR-455-5p/-3p	Ex vivo (clinical specimens) In vitro (C4-2, PC3, and DU145 cell lines)	PIR, LRP8, IGFBP3, DMBX1, CCDC64, TUBB1, KIF21B, and NFAM1		[114]
miR-3195, miR-3687, and miR-4417	Ex vivo (clinical samples) In vitro (PC3, LNCaP, VCAP, and DuCaP cell lines)	---	Lipofectamine RNAiMAX transfection reagent (Life Technologies, Waltham, MA, USA)	[115]
miR-375 and miR-301a	In vivo (Patient-derived xenograft models) In vitro (LNCaP and C42B cell lines)	---		[116]
miR-8080	Ex vivo (22Rv1, VCaP, and PCai1 cell lines) In vivo (Xenograft models) In Vitro (LNCaP cell line)	AR-V7	Lipofectamine 3000 (Thermo Fisher Scientific)	[117]
miR-128 mirVana	In vivo (Xenograft models) In vitro (LNCaP, DU145, PPC-1, and PC3 cell lines)	BMI-1	Lipofectamine RNAiMAX (Invitrogen)	[118]
miR-193b	Ex vivo (Clinical samples) In vivo (Xenograft models) In Vitro (22Rv1 cell line)	Cyclin D1	INTERFERin transfection reagent (Polyplus-transfection, Illkirch, France)	[119]
mirVana miR-124	In vivo (Xenograft models) In vitro (LNCaP, C4-2B, and 22Rv1 cell lines)	EZH2, Src, AR-V4, and AR-V7	Polyethylenimine (In vivo jetPEI)	[120]
miR-181-5p	In vivo (Xenograft models) In vitro (PC3 cell line)	GATA6	Lipofectamine 3000 regent (L3000015; Invitrogen, Carlsbad, CA, USA)	[121]
miR-30b	In vivo (Xenograft models) In vitro (VCaP and PC3 cell lines)	ERG	BLOCK-iT™ inducible H1 RNAi entry vector (Invitrogen)	[122]
miR-542-3p (lentivirus)	In vivo (Xenograft models) In vitro (DU145 cell line)	NOP2	HiPerFect transfection reagent (Qiagen)	[123]
miR-494 adenovirus	In vivo (Xenograft models) In vitro (PC3 cell line)	survivin	Polybrene (Sigma-Aldrich, St. Louis, MO, USA)	[124]
miR-193a-5p	Ex vivo (clinical samples) In vivo (Xenograft models) In vitro (PC3 cell line)	Bach2	Lipofectamine 2000 (Invitrogen)	[125]
miR-32	In vivo (Xenograft models) In vitro (C4-2 and LNCaP cell lines)	---	Lipofectamine 2000 (Invitrogen, Grand Island, NY, USA)	[126]
miR-421	In vivo (Xenograft models) In vitro (LNCaP/N-Myc and C4-2 cell lines)	---	Lipofectamine RNAiMAX (Invitrogen)	[127]
miR-217 and miR-181b-5p	In vitro (PC3 cell line)	---	DharmaFECT-1 transfection reagent	[128]
miR-212 and miR-22	Ex vivo (clinical specimens) In vivo (Xenograft models) In vitro (C4-2B cell line)	hnRNPH1		[129]
miR-513a-5p	In vivo (Xenograft models) In vitro (EnzS1-C4-2 and EnzR1-C4-2 cell lines)	---	Calcium chloride	[130]
miR-421	In vitro (22RV1 cell line)	---		[131]
miR-17	Ex vivo (clinical samples) In vitro (LNCaP and PC3 cell lines)	---		[132]
miR-205	Ex vivo (clinical samples) In vivo In vitro (LNCaP cell line)	SQLE	Polybrene (Sigma)	[133]
miR-196b	In vivo (xenograft model) In vitro (PPC cells)	Meis2		[134]
miR-30b-3p and miR-30d-5p (810)	In vitro (LNCaP, LAPC4, and VCAP cell lines)	AR	Lipofectamine (Life Technologies; Grand Island, NY, USA)	[135]
miR-34c	In vitro (LNCaP and C4-2 cell lines)	---	Lipofectamine 2000 transfection reagent (Invitrogen, Carlsbad, CA, USA)	[136]
miR-34a	Ex vivo (patient samples) In vivo (Xenograft models) In vitro (PC-3 and PC-3PR cell lines)	JAG1 Notch1	Lipofectamine RNAiMAX reagent (Invitrogen)	[137]
miR-346, miR-361-3p, and miR-197-3p	In vitro (PC3, 22RV1, LNCaP, C42, DU145, and HEK293T cell lines)	---	Lipofectamine RNAiMAX reagent (Invitrogen)	[138]

**Table 3:** MiRNA inhibitor studies in the CRPC context.

miRNA Drug	Study Type	miRNA Target	Delivery Method	References
anti-miR-193a-5p	In vivo (Xenograft models) In vitro (PC3 cell line)	Bach2	Lipofectamine 2000 (Invitrogen)	[125]
miR-26a inhibitor	In vitro (PC3 and LNCaP cell lines)	---	Lipofectamine 3000 (Invitrogen)	[112]
Cy3-modified miR-222-3p inhibitor	Ex vivo (clinical samples) In vitro (LNCaP cell line)	mTOR	Lipofectamine 2000 (Invitrogen)	[113]
anti-miR-3195, anti-miR-3687, and anti-miR-4417	Ex vivo (clinical samples) In vitro (PC3, LNCaP, VCAP, and DuCaP cell lines)	---	Lipofectamine RNAiMAX transfection reagent (Life Technologies)	[115]
miR-106a~363 sponge, anti-miR-363 inhibitor	In vivo (Patient-derived xenograft models) In vitro (LNCaP and C42B cell lines)	---		[116]
miR-1205 inhibitor	Ex vivo (clinical samples) In vitro (DU145 and PC3 cell lines)	EGLN3	Lipofectamine RNAiMAX (Thermo Fisher Scientific)	[108]
miR-8080 inhibitor	Ex vivo (22Rv1, VCaP, and PCai1 cell lines) In vivo (Xenograft models) In Vitro (LNCaP cell line)	AR-V7	Lipofectamine 3000 (Thermo Fisher Scientific)	[117]
miR-146a inhibitor	In vivo (Xenograft models) In vitro (LNCaP and PC3 cell lines)	---	INTERFERin (Polyplus-Transfection SA, Illkirch, France)	[139]
mirVana miR-128 inhibitor	In vivo (Xenograft models) In vitro (LNCaP, DU145, PCC-1, and PC3 cell lines)	BMI-1	Lipofectamine RNAiMAX (Invitrogen)	[118]
miR-149-5p inhibitor	In vitro (C4-2 cells)	Akt1	Lipofectamine^®^ 2000 (Invitrogen; Thermo Fisher Scientific, Inc.)	[111]
miR-181-5p inhibitor	In vivo (Xenograft models) In vitro (PC3 cell line)	GATA6	Lipofectamine 3000 regent (L3000015; Invitrogen, Carlsbad, CA, USA)	[121]
anti-miR-30b	In vivo (Xenograft models) In vitro (VCaP and PC3 cell lines)	ERG	BLOCK-iT™ inducible H1 RNAi entry vector (Invitrogen)	[122]
miR-99a inhibitor	In vitro (LNCaP and CB cell lines)	---	Lipofectamine^®^ RNAiMAX transfection reagent (Life Technologies Ltd., Paisley, UK)	[140]
miR-100 inhibitor	In vitro (LNCaP and CB cell lines)	---	Lipofectamine^®^ RNAiMAX transfection reagent (Life Technologies Ltd., Paisley, UK)	[140]
miR-193a-5p inhibitor	Ex vivo (clinical specimens) In vivo (Xenograft models) In vitro (PC3 cell line)	Bach2	Lipofectamine 2000 (Invitrogen)	[125]
miR-32 inhibitor	In vivo (Xenograft models) In vitro (C4-2 and LNCaP cell lines)	---	Lipofectamine 2000 (Invitrogen, Grand Island, NY, USA)	[126]
AMO-miR421	In vivo (Xenograft models) In vitro (LNCaP/N-Myc and C4-2 cell lines)	---	Lipofectamine RNAiMAX (Invitrogen)	[127]
miR-181a inhibitor	In vitro (C4-2B, C4-2B (docetaxel resistant), DU145, DU145 (docetaxel resistant), and PC3 cell lines)	---	Lipofectamine 2000 (Invitrogen)	[109]
miR-30a inhibitor	In vivo (Xenograft models) In vitro (LNCaP cell line)	MYBL2, FOXD1, and SOX4	Hiperfect transfection reagent (Qiagen)	[110]
miR-128 inhibitor miR-101 inhibitor	Ex vivo (clinical specimens) In vivo (Xenograft models) In vitro (DU145 cell line)	IFIT5	Xfect reagent (Clontech, San Jose, CA, USA) EZ Plex transfection reagent (EZPLEX)	[141]
anti-miR-421	In vitro (22RV1 cell line)	---		[131]
miR-196b inhibitor	In vivo (xenograft model) In vitro (PPC cells)	Meis2		[134]
miR-26a-5p inhibitor miR-101-3p inhibitor let-7a-5p inhibitor let-7b-5p inhibitor let-7c-5p inhibitor	In vivo (xenograft model) In vitro (LNCaP and 22Rv1 cell lines)	EZH2	Lipofectamine 2000 (Invitrogen)	[142]
miR-30b-3p inhibitor miR-30d-5p inhibitor	In vitro (LNCaP, LAPC4, and VCAP cell lines)	AR	Lipofectamine (Life Technologies; Grand Island, NY, USA)	[135]
miR-34c inhibitor	In vitro (LNCaP and C4-2 cell lines)	---	Lipofectamine 2000 transfection reagent (Invitrogen, Carlsbad, CA, USA)	[136]
miR-34a inhibitor	Ex vivo (patient samples) In vivo (Xenograft models) In vitro (PC-3 and PC-3PR cell lines)	JAG1 Notch1	Lipofectamine RNAiMAX reagent (Invitrogen)	[137]
miR-346, mimic miR-361-3p mimic and miR-197-3p mimic	In vitro (PC3, 22RV1, LNCaP, C42, DU145, and HEK293T cell lines)	---	Lipofectamine RNAiMAX reagent (Invitrogen)	[138]

## Data Availability

Not applicable.

## Supplementary Materials

The following supporting information can be downloaded at https://www.mdpi.com/article/10.3390/pharmaceutics16111347/s1: Supplementary Table S1: MiRNA mimic studies in the CRPC context; Supplementary Table S2: MiRNA inhibitor studies in the CRPC context.
