# Supplementary material for: MicroRNAs as Promising Therapeutic Agents Against Prostate Cancer Resistant to Castration—Where Are We Now?
Source: Pharmaceutics. 2024 Oct 22;16(11):1347. doi: 10.3390/pharmaceutics16111347 (PMC11597238; doi:10.3390/pharmaceutics16111347)
Supplement: Supplementary file 1 [file pharmaceutics-16-01347-s001.zip › pharmaceutics-3213573-supplementary.pdf]

pharmaceutics

MDPI

**Supplementary Material:**

**Supplementary Table S1:** miRNAs mimics studies in CRPC context.

| miRNA Drug | Targeted miRNA | miRNA Expression | Main Results | References |
|---|---|---|---|---|
| miR-1247–5p | miR-1247-5p | Up regulated in CRPC | The miR-1247-5p is over-expressed in CRPC and targets MYCBP2, making it a potential high-scoring target. | [107] |
| miR-218 , miR-145 , miR-197 , miR-149 , miR-122, let-7b | miR-218, miR-145, miR-197, miR-122, let-7b | miR-218, miR-197- up-regulated in CRPC<br>miR-145, miR-122, let-7b - down-regulated in CRPC | miR-122, let-7b, miR-218, miR-197, miR-145 and miR-149 may play a role in CRPC development by affecting Ras, Rho proteins and the SCF complex. | [108] |
| miR-1205 | miR-1205 | Up regulated in CRPC | miR-1205 is overexpressed in castration-resistant prostate cancer and its oncogenic role through EGLN3 targeting suggests a potential contribution to the genetic risk of CRPC. | [109] |
| miR-181a | miR-181a | Up regulated in docetaxel resistant cells | Docetaxel-resistant prostate cancer cell lines show a significant increase in miR-181a expression. Knockdown of miR-181a restores sensitivity to docetaxel in resistant cells. Overexpression of miR-181a confers resistance in both resistant and sensitive cells, probably through modulation of p53 phosphorylation. | [110] |
| miR-30a | miR-30a | Downregulated in CRPC | miR-30a inhibit androgen-independent growth of PC, by directly targeting SOX4, MYBL2, and FOXD1. | [111] |
| miR-149 | miR-149 | Downregulated in CRPC | Targeting Akt1 expression through miR-149 overexpression represents a potential novel therapeutic approach for CRPC. | [112] |
| miR-26a | miR-26a | miR-26a expression levels were higher in LNCAP cells compared with PC-3 cells | Exosomal miR-26a could be used as a personalized delivery vehicle for the targeted delivery of therapeutic agents to prostate cancer cells and tissues. | [113] |
| Cy3-modified miR-222-3p | Exosomal miR-222-3p | miR-222-3p expression is elevated in androgen-independent prostate cancer cells | Exos-miR-222-3p, by targeting MIDN, promotes the transformation of androgen dependent PC cells into androgen independent PC-like cells, in part by triggering mTOR signaling. | [114] |
| miR-455-5p/-3p | miR-455-5p/-3p | miR-455-5p and miR-455-3p expression is downregulated in PCa tissues compared with benign prostate tissues. PCa cell lines also had very low expression levels of both miRNAs | miR-455-duplex act as antitumor miRNAs in PCa cells. PIR expression is directly regulated by miR-455-5p and might be a promising diagnostic marker for HSPC and CRPC. | [115] |

| miRNA Drug | Targeted miRNA | miRNA Expression | Main Results | References |
|---|---|---|---|---|
| miR-3195<br>miR-3687<br>miR-4417 | miR-3195, miR-3687<br>miR-4417 | Up regulated in CRPC | miR-205 and miR-92b are downregulated and miR-3195, miR-3687 and miR-4417 are upregulated in CRPC. High levels of miR-3687 decrease cell migration and invasion, while high levels of miR-3195 increase cell migration. | [116] |
| miR-375<br>miR-301a | miR-375<br>miR-301a | Up regulated in PCa cell lines | The miR-106a~363 cluster and other miRNA changes that promote neuroendocrine prostate cancer (NEPC) could potentially be used to develop effective targeted therapies for NEPC. | [117] |
| miR-8080 | miR-8080 | --- | The upregulation of miR-8080 by luteolin plays a critical role in reducing AR-V7 protein levels, inhibiting tumorigenesis and overcoming enzalutamide resistance in CRPC. | [118] |
| miR-128 mirVana | miR-128 | miR128 levels were reported to be downregulated in prostate cancer and their metastases | The tumour suppressive effects of miR-128 are linked to its ability to inhibit cancer stem cells (CSCs) by targeting key molecules such as BMI-1. | [119] |
| miR-193b | miR-193b | -- | miR-193b targets cyclin D1 in prostate cancer. | [120] |
| mirVana miR-124 | miR-124 | miR-124 levels were reported to be downregulated in clinical PCa samples | miR-124 regulates the oncopathway in PCa by targeting EZH2, Src and AR variants, thus contributing to pathogenesis and treatment resistance. Restoring miR-124 inhibits the growth of enzalutamide-resistant PCa cells and increases their sensitivity to enzalutamide. | [121] |
| miR-181-5p | miR-181-5p | miR-181-5p levels were reported to be downregulated in PCa patients | Niraparib inhibits prostate cancer cell proliferation and metastasis as well as tumour growth in mice by modulating the MEG3/miR-181-5p/GATA6 lncRNA pathway. | [122] |
| miR-30b | miR-30b | miR-30 is downmodulated in prostate cancer cells, especially at the metastatic stage. | The study identifies a new post-transcriptional mechanism by which Src and growth signals regulate TMPRSS2-ERG through miR-30. | [123] |
| miR-542-3p (lentivirus) | miR-542-3p | miR-542-3p expression levels were lower in DU145 and PC3 cells compared with RWPE-1 cells | LINC00963 promotes the metastasis of prostate cancer by binding to miR-542-3p, which in turn increases the expression of NOP2. | [124] |
| miR-494 adenovírus | miR-494 | miR-494 is lower in PCa than in normal prostate tissue | Using different strategies to inhibit survivin may help to improve the effectiveness of treating PCa. | [125] |
| miR-193a-5p | miR-193a-5p | miR-193a-5p levels were reported to be upregulated in PC tissues and PC cell lines | Inhibition of miR-193a-5p or disruption of the miR-193a-5p-Bach2-HO-1 pathway may represent a new therapeutic strategy for CRPC | [126] |
| miR-32 | miR-32 | --- | Enzalutamide (or Casodex) and infiltrating mast cells enhance PCa cell recruitment of more mast cells, potentially leading to increased PCa NE differentiation via modulation of miRNA32 signalling. | [127] |
| miR-421 | miR-421 | --- | N-Myc regulates the miR-421/ATM pathway differentially, contributing to ADT and enzalutamide resistance, and that combined use of an ATM inhibitor and enzalutamide could re-sensitise N-Myc overexpressing CRPC cells. | [128] |
| miR-217<br>miR-181b-5p | miR-217<br>miR-181b-5p | --- | miR-217 and miR-181b-5p mimic increased the efficacy of docetaxel and cabazitaxel in CRPC in vitro | [129] |

| miRNA Drug | Targeted miRNA | miRNA Expression | Main Results | References |
| --- | --- | --- | --- | --- |
| miR-212<br>miR-22 | miR-212 | Downregulated in CRPC | Targeted disruption of the hnRNPH1-AR axis might improve clinical outcomes in patients with advanced prostate cancer, particularly in African American men | [130] |
| miR-513a-5p | miR-513a-5p | --- | High-dose androgen with PD-1/PD-L1 checkpoint inhibitors may help suppress CRPC progression. | [131] |
| miR-421 | miR-421 | --- | MALAT1 shields prostate cancer tumour cells from anticancer drugs by initiating the HR pathway | [132] |
| miR-17 | miR-17 | --- | cirITCH functions as a tumour suppressor in prostate cancer cells in an AR-independent manner through the Wnt/β-catenin and PI3K/AKT/mTOR pathways. | [133] |
| miR-205 | miR-205 | Downregulated in CRPC | SQLE is a target of miR-205 in PCa and a crucial driver of disease progression and malignancy through regulation of cholesterol biosynthesis and AR activation. | [134] |
| miR-196b | miR-196b | --- | The study provides a baseline for the development of highly effective therapeutic strategies for CRPC that avoid the indiscriminate inhibition of IKK/NF-kB in normal cells. | [135] |
| miR-30b-3p<br>miR-30d-5p<br>(810) | miR-30b-3p<br>miR-30d-5p | miR-30c-5p and miR-30d-5p levels were significantly lower in CRPC compared to healthy prostate tissues | This study provides a large evaluation of AR-regulating miRNAs in PCa | [136] |
| miR-34c | miR-34C | --- | This study suggests that miR-34C may play an essential role in conferring castration resistance by stabilizing the PSCS population. | [137] |
| miR-34a | miR-34a | miR-34a was down-regulated in PC-3 paclitaxel resistant cells compared with PC-3 cells | miR-34a is involved in the development of chemosensitivity to paclitaxel. | [138] |
| miR-346,<br>miR--361-3p<br>and miR-197-3p | miR-346,<br>miR--361-3p<br>and miR-197-3p | --- | The study identified miRs that modulate AR activity in PC and CRPC. | [139] |

**Supplementary Table S2:** miRNAs inhibitors studies in CRPC context.

| miRNA Drug | Targeted miRNA | miRNA Expression | Main Results | References |
|---|---|---|---|---|
| anti-miR-193a-5p | miR-193a-5p | Up regulated in PC tissues and PC cell lines | Inhibition of miR-193a-5p or disruption of the miR-193a-5p-Bach2-HO-1 pathway may represent a promising new therapeutic strategy for CRPC. | [126] |
| miR-26a inhibitor | miR-26a | miR-26a expression levels were higher in LNCAP cells compared with PC-3 cells | Exosomal miR-26a could be used as a personalized delivery vehicle for the targeted delivery of therapeutic agents to prostate cancer cells and tissues. | [113] |
| Cy3-modified miR-222-3p inhibitor | Exosomal miR-222-3p | miR-222-3p expression is elevated in androgen-independent prostate cancer cells | Exos-miR-222-3p, by targeting MIDN, promotes the transformation of androgen dependent PC cells into androgen independent PC-like cells, in part by triggering mTOR signaling. | [114] |
| anti-miR-3195<br>anti- miR-3687<br>anti- miR-4417 | miR-3195,<br>miR-3687<br>miR-4417 | Up regulated in CRPC | miR-205 and miR-92b are downregulated and miR-3195, miR-3687 and miR-4417 are upregulated in CRPC. High levels of miR-3687 decrease cell migration and invasion, while high levels of miR-3195 increase cell migration. | [116] |
| miR-106a~363 sponge<br>anti-miR-363 inhibitor | miR-106a~363<br>miR-363 | miR-363 and miR-106a are downregulated in CRPC-NE | The miR-106a~363 cluster and other miRNA changes that promote neuroendocrine prostate cancer (NEPC) could potentially be used to develop effective targeted therapies for NEPC. | [117] |
| miR-1205 inhibitor | miR-1205 | miR-1205 is upregulated in both CRPC cell lines and in primary tumors | miR-1205 contributes to the genetic risk of CRPC acting as an oncogene through its targeting of EGLN3. | [109] |
| miR-8080 inhibitor | miR-8080 | --- | The upregulation of miR-8080 by luteolin plays a critical role in reducing AR-V7 protein levels, inhibiting tumorigenesis and overcoming enzalutamide resistance in CRPC. As a result, miR-8080 is emerging as a promising therapeutic target for CRPC. | [118] |
| miR-146a inhibitor | miR-146a | Up regulated in CRPC | Increasing miR-146a expression by hypomethylating the miR-146a promoter with 5-Aza-CdR was associated with slower progression of castration-resistant prostate cancer. | [140] |
| mirVana miR-128 inhibitor | miR-128 | miR128 levels were reported to be downregulated in prostate cancer and their metastases | The tumour suppressive effects of miR-128 are linked to its ability to inhibit cancer stem cells (CSCs) by targeting key molecules such as BMI-1. | [119] |
| miR-149-5p inhibitor | miR-149-5p | --- | miR-149 acts as a tumour suppressor in the C4-2 CRPC cell line, inhibiting progression via the AR signalling pathway by targeting Akt1. | [112] |
| miR-181-5p inhibitor | miR-181-5p | miR-181-5p levels were reported to be downregulated in PCa patients | Niraparib inhibits prostate cancer cell proliferation and metastasis as well as tumour growth in mice by modulating the MEG3/miR-181-5p/GATA6 lncRNA pathway. | [122] |
| anti-miR-30b | miR-30b | miR-30 is downmodulated in prostate cancer cells, especially at the metastatic stage. | The study identifies a new post-transcriptional mechanism by which Src and growth signals regulate TMPRSS2-ERG through miR-30. | [123] |
| miR-99a inhibitor | miR-99a | Downregulated in CRPC | miR-99 family can act as markers of radiation sensitivity and as potential therapeutic targets to improve the efficiency of radiotherapy. | [141] |
| miR-100 inhibitor | miR-100 | Downregulated in CRPC | miR-99 family can act as markers of radiation sensitivity and as potential therapeutic targets to improve the efficiency of radiotherapy. | [141] |

| miRNA Drug | Targeted miRNA | miRNA Expression | Main Results | References |
|---|---|---|---|---|
| miR-193a-5p inhibitor | miR-193a-5p | miR-193a-5p levels were reported to be up-regulated in PC tissues and PC cell lines | Inhibition of miR-193a-5p or disruption of the miR-193a-5p-Bach2-HO-1 pathway may represent a new therapeutic strategy for CRPC | [126] |
| miR-32 inhibitor | miR-32 | --- | Enzalutamide (or Casodex) and infiltrating mast cells enhance PCa cell recruitment of more mast cells, potentially leading to increased PCa NE differentiation via modulation of miRNA32 signalling. | [127] |
| AMO-miR421 | miR-421 | --- | N-Myc regulates the miR-421/ATM pathway differentially, contributing to ADT and enzalutamide resistance, and that combined use of an ATM inhibitor and enzalutamide could re-sensitise N-Myc overexpressing CRPC cells. | [128] |
| miR-181a inhibitor | miR-181a | --- | Docetaxel-resistant prostate cancer cell lines show a significant increase in miR-181a expression. Knockdown of miR-181a restores sensitivity to docetaxel in resistant cells. Overexpression of miR-181a confers resistance in both resistant and sensitive cells, probably through modulation of p53 phosphorylation. | [110] |
| miR-30a inhibitor | miR-30a | Downregulated in CRPC | miR-30a inhibit androgen-independent growth of PC, by directly targeting SOX4, MYBL2, and FOXD1. | [111] |
| miR-128 inhibitor<br>miR-101 inhibitor | miR-128<br>miR-101 | --- | STAT1-IFIT5 plays a crucial role in PCSC acquisition | [142] |
| anti-miR-421 | miR-421 | --- | MALAT1 shields prostate cancer tumor cells from anticancer drugs by initiating the HR pathway. | [132] |
| miR-196b inhibitor | miR-196b | --- | The study provides a baseline for the development of highly effective therapeutic strategies for CRPC that avoid the indiscriminate inhibition of IKK/NF-kB in normal cells. | [135] |
| miR-26a-5p inhibitor<br>miR-101–3p inhibitor<br>let-7a-5p inhibitor<br>let-7b-5p inhibitor<br>let-7c-5p inhibitor | miR-26a-5p<br>miR-101–3p<br>let-7a-5p<br>let-7b-5p<br>let-7c-5p | --- | The combination of GSK126 and metformin would be an effective approach for future PCa therapy | [143] |
| miR-30b-3p inhibitor<br>miR-30d-5p inhibitor | miR-30b-3p<br>miR-30d-5p | miR-30c-5p and miR-30d-5p levels were significantly lower in CRPC compared to healthy prostate tissues | This study provides a large evaluation of AR-regulating miRNAs in PCa | [136] |
| miR-34c inhibitor | miR-34C | --- | This study suggests that miR-34C may play an essential role in conferring castration resistance by stabilizing the PSCS population. | [137] |
| miR-34a inhibitor | miR-34a | miR-34a was down-regulated in PC-3 paclitaxel resistant cells compared with PC-3 cells | miR-34a is involved in the development of chemosensitivity to paclitaxel. | [138] |

| miRNA Drug | Targeted miRNA | miRNA Expression | Main Results | References |
|---|---|---|---|---|
| miR-346, mimic miR--361-3p mimic and miR-197-3p mimic | miR-346, miR--361-3p and miR-197-3p | --- | The study identified miRs that modulate AR activity in PC and CRPC. | [139] |
